# The Selector Gene *apterous* and Notch Are Required to Locally Increase Mechanical Cell Bond Tension at the *Drosophila* Dorsoventral Compartment Boundary

**DOI:** 10.1371/journal.pone.0161668

**Published:** 2016-08-23

**Authors:** Marcus Michel, Maryam Aliee, Katrin Rudolf, Lisa Bialas, Frank Jülicher, Christian Dahmann

**Affiliations:** 1 Institute of Genetics, Technische Universität Dresden, 01062, Dresden, Germany; 2 Max Planck Institute for the Physics of Complex Systems, Nöthnitzer Strasse 38, 01187, Dresden, Germany; University of Dayton, UNITED STATES

## Abstract

The separation of cells with distinct fates and functions is important for tissue and organ formation during animal development. Regions of different fates within tissues are often separated from another along straight boundaries. These compartment boundaries play a crucial role in tissue patterning and growth by stably positioning organizers. In *Drosophila*, the wing imaginal disc is subdivided into a dorsal and a ventral compartment. Cells of the dorsal, but not ventral, compartment express the selector gene *apterous*. Apterous expression sets in motion a gene regulatory cascade that leads to the activation of Notch signaling in a few cell rows on either side of the dorsoventral compartment boundary. Both *Notch* and *apterous* mutant clones disturb the separation of dorsal and ventral cells. Maintenance of the straight shape of the dorsoventral boundary involves a local increase in mechanical tension at cell bonds along the boundary. The mechanisms by which cell bond tension is locally increased however remain unknown. Here we use a combination of laser ablation of cell bonds, quantitative image analysis, and genetic mutants to show that Notch and Apterous are required to increase cell bond tension along the dorsoventral compartment boundary. Moreover, clonal expression of the Apterous target gene *capricious* results in cell separation and increased cell bond tension at the clone borders. Finally, using a vertex model to simulate tissue growth, we find that an increase in cell bond tension at the borders of cell clones, but not throughout the cell clone, can lead to cell separation. We conclude that Apterous and Notch maintain the characteristic straight shape of the dorsoventral compartment boundary by locally increasing cell bond tension.

## Introduction

The specification of cell fate is important for organizing cells into functional tissues during animal development. The separation of cells with different fates and functions by boundaries is a prominent example of tissue organization [1,2,3,4,5]. Signaling between cells with different fates sets up a local source of organizers along compartments. Signaling molecules emanating from these organizer regions influence cell fate and growth of the entire tissue. Compartment boundaries thus serve as a reference line during pattern formation and growth [6]. Compartments have been identified in vertebrates and invertebrates. In vertebrates, for example the embryonic hindbrain, telencephalon and limb primordia are subdivided into compartments [1,2]. In *Drosophila*, the wing primordium (wing imaginal disc) is subdivided by two compartment boundaries. The anteroposterior compartment boundary (AP boundary) is established during embryonic development and separates cells of anterior and posterior fates in the wing primordium [7]. The dorsoventral compartment boundary (DV) boundary arises only during early-third instar larval development and separates dorsal and ventral cells [8,9]. In vertebrates, the establishment of compartment boundaries requires signaling between neighboring compartments. For example, signaling by the family of Eph receptor tyrosine kinases and their ephrin ligands plays a central role for establishing and maintaining compartment boundaries [1]. In *Drosophila*, both signaling between compartments and the compartment-specific expression of selector genes is required for the establishment and maintenance of compartment boundaries. Hedgehog, Dpp and Eph signaling, in addition to the selector genes *engrailed* and *invected*, are required to maintain the characteristic straight shape of the AP boundary [10,11,12,13,14,15,16]. Maintenance of a proper DV boundary shape requires the selector gene *apterous* and Notch signaling [17,18,19]. Apterous is expressed in all cells of the dorsal compartment [20,21,22]. Loss of Apterous activity transforms dorsal cells into a ventral fate [21]. Apterous, a LIM-domain containing transcription factor [23], induces expression of several target genes in dorsal cells. During mid-third instar larval development, for example, Apterous induces expression of the two leucine-rich repeat proteins Capricious and Tartan that have been proposed to be involved in maintaining the straight shape of the DV boundary [24]. Apterous also induces expression of Fringe, a glycosyltransferase that modifies several EGF domains in the extracellular region of the Notch receptor [25,26]. This glycosylation alters the interaction of Notch with its ligands Delta and Serrate in a way that Delta signaling is enhanced while Serrate signaling is suppressed. As a consequence, Notch signal transduction is activated in approximately 2–4 cell rows on either side of the DV boundary. Notch signal transduction in cells along the DV boundary induces expression of Wingless, which in turn contributes to wing disc patterning and growth [27]. Clones of cells mutant for *apterous* or *Notch* disturb the shape of the DV boundary [17,18], showing that Apterous and Notch play important roles in separating dorsal and ventral cells along this compartment boundary.

We have previously shown that two physical mechanisms can account for the shape of the DV boundary: First, global tension anisotropies in the wing imaginal disc that result in cell elongation and consequently oriented cell division and, second, a local increase in mechanical tension at adherens junctions (termed cell bond tension) along the compartment boundary [28]. Cell bond tension is generated by actomyosin contractility and cell-cell adhesion. Local increases in cell bond tension bias cell re-arrangements in a way that cells from neighboring compartments are kept separated [29]. Moreover, recent data show that cell bond tension at the AP boundary is locally increased in response to the difference in Hedgehog signal transduction activity present between anterior and posterior cells [14]. Engrailed, in addition to its role in modulating the Hedgehog signal transduction, also contributes to straight AP boundary shape, albeit seemingly without influencing cell bond tension [14]. The roles of Apterous and Notch for the local increase in cell bond tension along the DV boundary, however, remain unknown. Here we show that both Apterous and Notch are required to increase cell bond tension along this compartment boundary.

## Results

### The selector gene *apterous* is required to maintain the characteristic straight shape of the DV boundary

**Fig 1:**
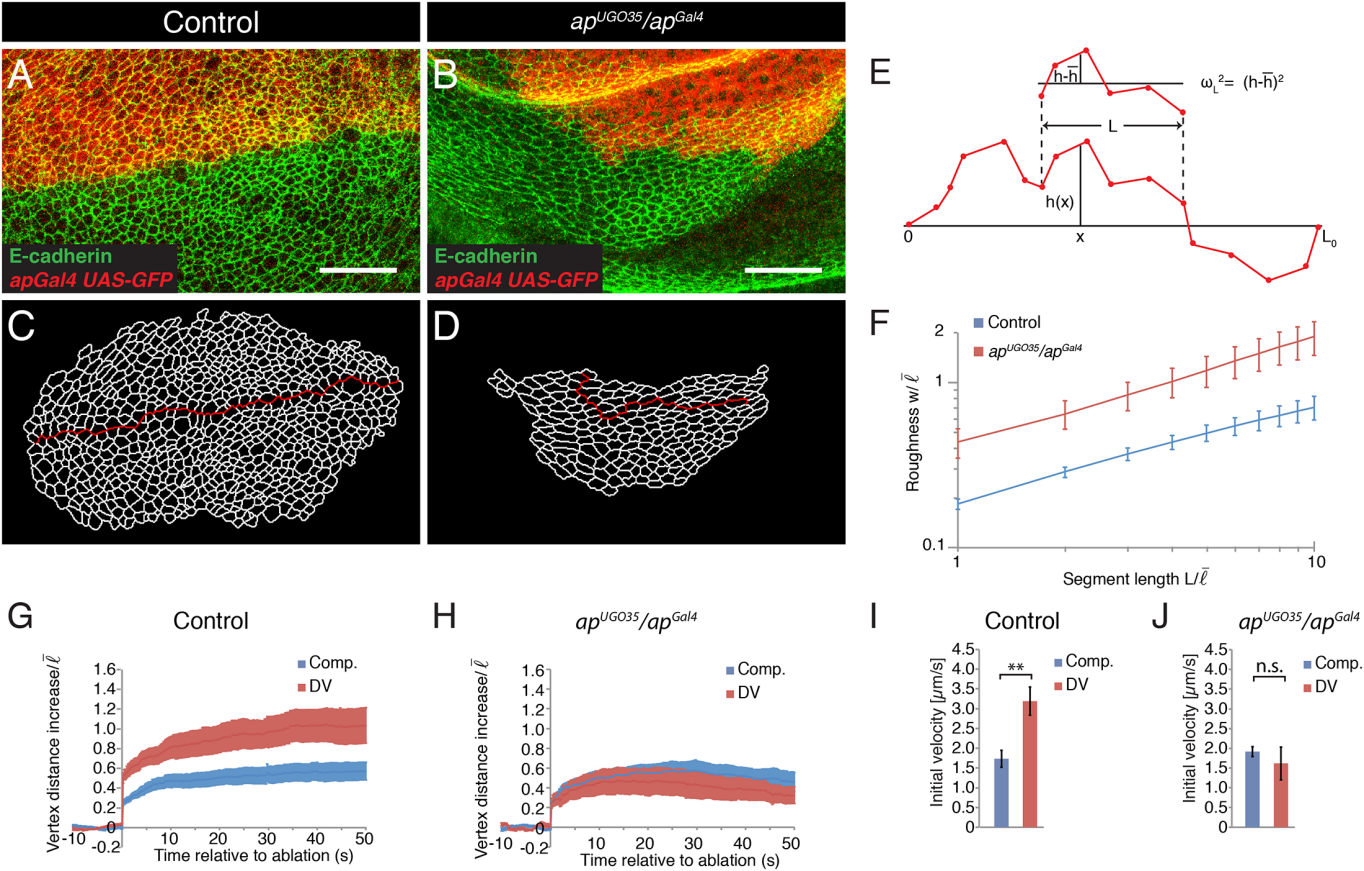
Apterous is required for the characteristic straight shape of the DV boundary and for the local increase in cell bond tension. (A,B) Wing discs from *ap*^*Gal4*^ (control) and *ap*^*UGO35*^*/ap*^*Gal4*^ mutant larvae expressing GFP (red) in the dorsal compartment stained for E-cadherin (green) to visualize adherens junctions. Scale bars: 10 μm. (C,D) Segmentation of the images shown in (A) and (B). The red line marks the DV boundary. (E) Scheme depicting the measurement of roughness *w* of a boundary. The shape of the boundary is described by the function *h*(*x*) as the orthogonal distance of the boundary from the *x* axis, connecting the end points of the boundary, for any position x. For two points within the segment length *L* roughness is given by the average deviation from the mean value, ${\left(h\left(x\right)-\bar{h}\right)}^{2}$, where $\bar{h}$ is the average of *h*(*x*) within the segment length *L*. We then average roughness along the boundary for any *L* indicated. (F) Roughness *w* of the DV boundary for the segment lengths *L* for the genotypes indicated in (A,B). Segment length and roughness values are normalized by the average cell bond length in the tissue $\bar{l}$ = 1.7 μm. Mean and s.e.m. are shown (control, n = 6 wing discs; mutant, n = 7 wing discs). *P* <0.05 for *L* = 1–10. (G,H) Change in distance *d* between the vertices of cell bonds located within the compartments (comp.) or at the DV boundary after ablation (normalized to $\bar{l}$) as a function of time for wing discs of *ap*^*Gal4*^ (control) and *ap*^*UGO35*^*/ap*^*Gal4*^ mutant larvae. Mean and s.e.m. are shown (control, n = 10 comp., n = 11 DV; mutant, n = 9 comp., n = 6 DV cuts). (I,J) Initial velocity of vertex displacement after ablation of the indicated types of cell bonds for control (I) and *ap*^*UGO35*^*/ap*^*Gal4*^ mutant (J) larvae. Mean and s.e.m. are shown (n as in G,H). ** *P*<0.01; n.s. not significant.

To address the role of Apterous in shaping the DV boundary, we quantified and compared the shape of the DV boundary in control and *apterous* mutant wing discs. We used a combination of an *apterous* null allele (*ap*^*UGO35*^) and a hypomorphic *apterous* allele that was generated by inserting the yeast *Gal4* gene into the *apterous* locus (*ap*^*Gal4*^). The advantage of this allelic combination is that the *ap*^*Gal4*^ allele can be used to express transgenes in the dorsal compartment of the wing disc and thus to mark the DV boundary [24]. We refer in the following to the allelic combination of *ap*^*UGO35*^ / *ap*^*Gal4*^ as *apterous* mutant. Adherens junctions were identified by E-cadherin immunostainings and the DV boundary was visualized by expression of GFP under control of *ap*^*Gal4*^. As reported previously [24], the shape of the DV boundary was much more irregular in *apterous* mutant wing discs compared to controls (Fig 1A and 1B). To test whether Apterous influences DV boundary shape at a local cell-to-cell scale or at a global tissue scale, we quantitatively analyzed the shape of the DV boundary in *apterous* mutants. We segmented the adherens junctional network in the acquired images and identified the cell bonds along the DV boundary (Fig 1C and 1D). The shape of the DV boundary was characterized by a geometric measure termed roughness *w* [28,30]. Roughness *w* characterizes the variance of the distance of a boundary from a straight line along boundary segments of varying length. We display roughness w as a function of segment length *L* (Fig 1E). This geometric measure therefore determines the scaling behavior of the boundary shape. The roughness of the DV boundary was for all segment length *L* analyzed increased in wing discs of *apterous* mutants compared to controls (Fig 1F). These experiments reveal that Apterous is required to maintain the characteristic straight shape of the DV boundary both at local and global scales.

### The selector gene *apterous* is required to locally increase cell bond tension along the DV boundary

Cell bond tension is locally increased at the DV boundary compared to the bulk of the tissue [28]. It has been proposed that the local increase in cell bond tension is involved in maintaining the characteristic straight shape of the DV boundary [28,31,32]. Cell bond tension can be measured by ablating individual cell bonds and by subsequently quantifying the resulting displacement of the two ends of the ablated cell bonds [33]. The initial velocity of this vertex displacement is a relative measure of cell bond tension [34]. To test whether Apterous is required to increase cell bond tension along the DV boundary, we ablated single cell bonds in control and *apterous* mutant wing discs with a laser beam and compared the resulting vertex displacements. Final vertex displacement and initial velocity of vertex displacements resulting from ablating junctions within the dorsal or ventral compartment were indistinguishable between control and *apterous* mutant wing discs (Fig 1G–1J). Ablation of cell bonds along the DV boundary in control wing discs resulted in an approximately two-fold increase in initial velocity of vertex displacement (Fig 1I). However, in *apterous* mutant wing discs, ablations of cell bonds along the DV boundary no longer showed an increased initial velocity of vertex displacement (Fig 1J). These results show that Apterous is not required for generating cell bond tension within the bulk of the wing disc. Importantly, however, Apterous is required to increase cell bond tension along the DV boundary.

### Notch is required to maintain the characteristic straight shape of the DV boundary

**Fig 2:**
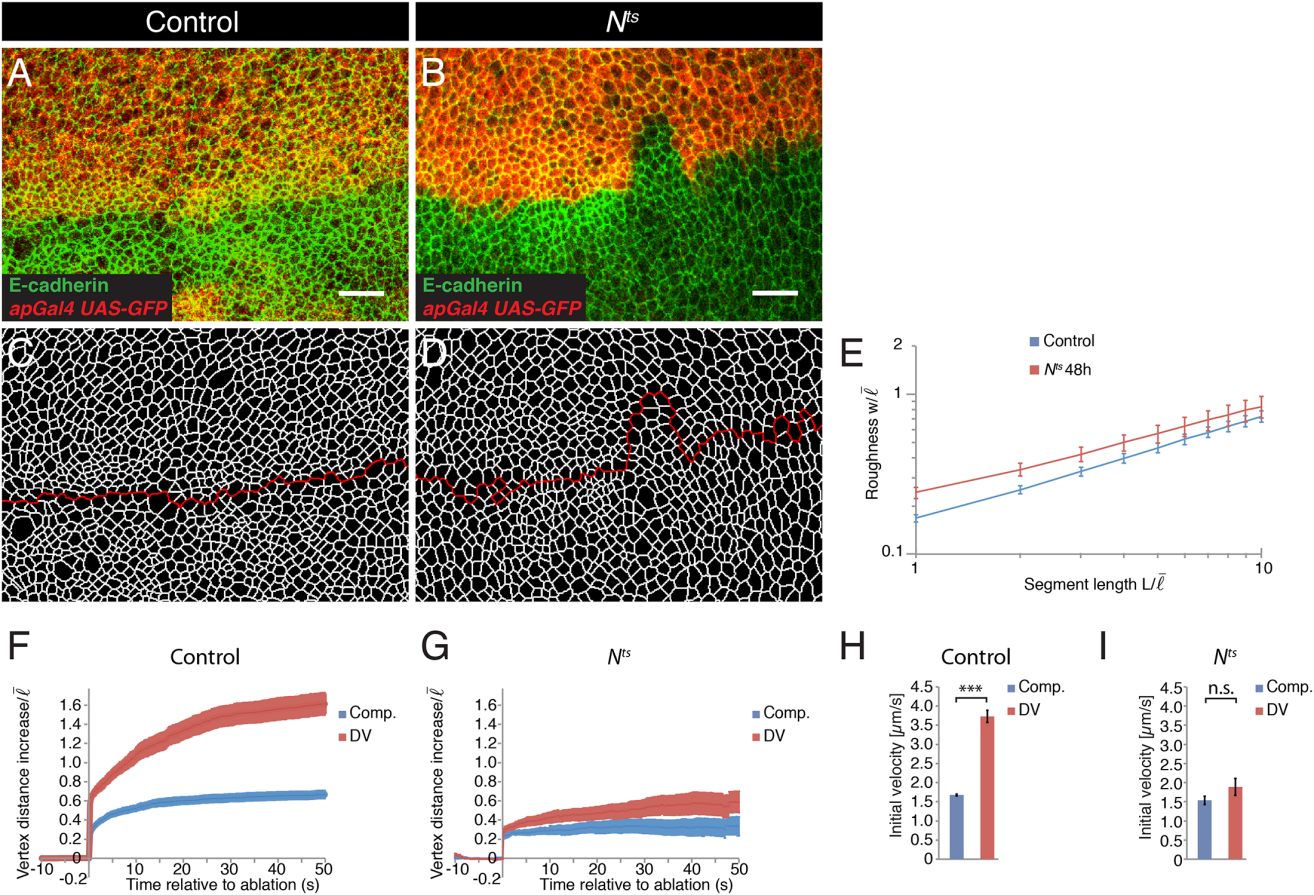
Notch is required for the characteristic straight shape of the DV boundary and for the local increase in cell bond tension. (A,B) Wing discs from control and *N*^*ts*^ mutant larvae reared for 48 h at 29°C and expressing GFP (red) in the dorsal compartment (*apGal4*, *UAS-GFP*) were stained for E-cadherin (green) to visualize adherens junctions. Scale bars: 10 μm. (C,D) Segmentation of the images shown in (A) and (B). The red line marks the DV boundary. (E) Roughness *w* of the DV boundary for the segment lengths *L* for the genotypes indicated in (A,B). Segment length and roughness values are normalized by the average cell bond length in the tissue $\bar{l}$ = 1.7 μm. Mean and s.e.m. are shown (control, n = 7 wing discs; mutant, n = 7 wing discs). *P* <0.01–0.05 for L = 1–2. (F,G) Change in distance *d* between the vertices of cell bonds located within the compartments (comp.) or at the DV boundary after ablation (normalized to $\bar{l}$) as a function of time for wing discs of control and *N*^*ts*^ mutant larvae reared for 48 h at 29°C. Mean and s.e.m. are shown (control, n = 14 comp., n = 11 DV; mutant, n = 9 comp., n = 10 DV cuts). (H,I) Initial velocity of vertex displacement after ablation of the indicated types of cell bonds for control (H) and *N*^*ts*^ mutant (I) larvae reared for 48 h at 29°C. Mean and s.e.m. are shown (n as in F,G). *** *P*<0.001; n.s. not significant.

Notch signaling is required for separating dorsal and ventral cells along the DV boundary [18,19,35,36]. To test the role of Notch in maintaining the straight shape of the DV boundary, we quantified the roughness *w* of the DV boundary in control and *N*^*ts*^ mutant wing discs. *N*^*ts*^ is a temperature-sensitive allele of *N* that results in a severe reduction of Notch activity when animals are reared at the restrictive temperature (S1A and S1B Fig). When larvae were raised for 48 hours at restrictive temperature, the shape of the DV boundary was more irregular in mutant wings discs compared to controls [36] (Fig 2A–2D). Moreover, the roughness w of the DV boundary was increased in *N*^*ts*^ mutant wing discs compared to the roughness of the DV boundary in control wing discs for all segment length *L* along the boundary analyzed (Fig 2E). These data show that Notch is required to maintain the characteristic straight shape of the DV boundary.

### Notch is required to locally increase cell bond tension along the DV boundary

F-actin and Myosin II levels are transiently elevated at cell bonds along the DV boundary during mid-third instar larval development [31,36]. Increased levels of F-actin and Myosin II require activation of Notch [31,36]. Cell bond tension is increased along the DV boundary during mid and late third instar larval development, even though an increase in F-actin and Myosin II levels at the DV boundary are no longer detectable during late third instar stage [28]. To test whether Notch is required for the increased cell bond tension along the DV boundary in late third instar wing discs, we ablated single cell bonds and quantified the resulting vertex displacements. In larvae reared for 48 hours at restrictive temperature, vertex displacements resulting from ablating cell bonds within the dorsal or ventral compartment were indistinguishable between control and *N*^*ts*^ mutant wing discs (Fig 2F–2I). Ablation of cell bonds along the DV boundary in control wing discs resulted in an approximately two-fold increase in initial velocity of vertex displacement (Fig 2H). However, in *N*^*ts*^ mutant wing discs, ablations of cell bonds along the DV boundary no longer showed an increased final vertex displacement and initial velocity of vertex displacement (Fig 2I). Thus, similar to Apterous, Notch is not required for generating cell bond tension in the bulk of the tissue, yet it is required for the local increase in cell bond tension along the DV boundary.

### Expression of N^intra^ in clones of cells reduces clonal roughness

**Fig 3:**
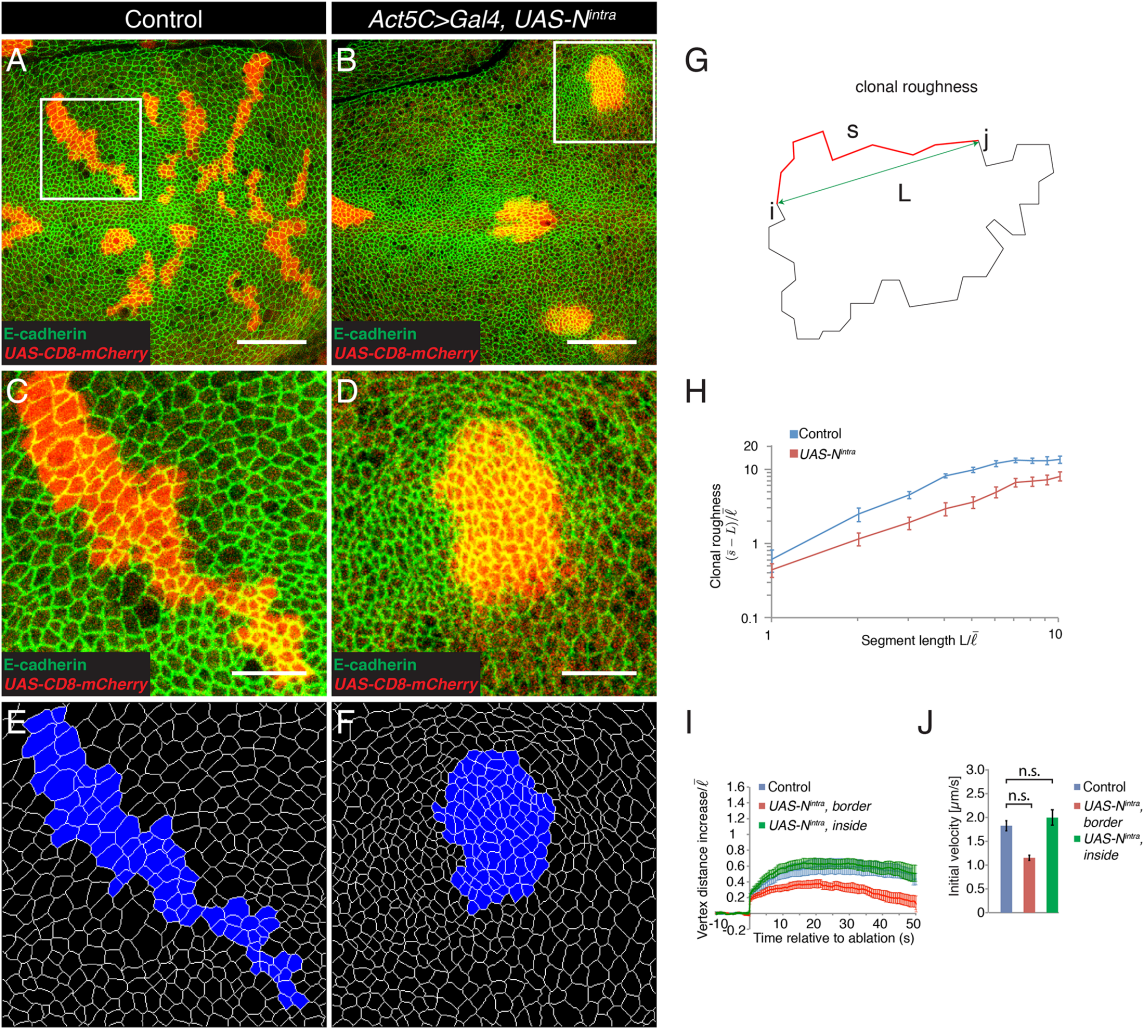
Expression of N^intra^ in clones of cells leads to smooth clone borders but no change in cell bond tension at the clone border. (A,B) Wing discs of wandering stage third instar larvae displaying control clones (A) or clones expressing N^intra^ (B, *Act5C>Gal4*, *UAS-N*^*intra*^) marked by the expression of CD8-mCherry (red) and stained for E-cadherin (green). Scale bars: 30 μm. (C,D) Magnification of the inset in (A,B). Scale bars: 10 μm. (E,F) Segmentation of the images shown in (C) and (D). The clones are marked in blue. (G) Scheme depicting the measurement of clonal roughness. The shape of a clone is described by a sequence of the position of the vertices along the clone border *R*_*i*_ = (*X*_*i*_,*Y*_*i*_). For any two vertices i and j at the clone border, *s* is the contour length along the clone border and *L* is their point-to-point segment length. For any indicated segment length *L*, clonal roughness measures the average of deviation of contour length from a straight line, $\bar{s}-L$. (H) Clonal roughness for control clones and clones expressing N^intra^. Segment length and clonal roughness values are normalized by the average cell bond length in the tissue $\bar{l}$ = 1.7 μm. Mean and s.e.m. are shown (control, n = 9 wing discs; mutant, n = 8 wing discs), *P* <0.001–0.05 for L = 2–10. (I) Change in distance *d* between the vertices at borders of control clones or of clones expressing N^intra^ or of vertices inside clones expressing N^intra^ after ablation (normalized to $\bar{l}$) as a function of time. Mean and s.e.m. are shown (control, n = 9; N^intra^, n = 9; inside N^intra^, n = 6 cuts). (J) Initial velocity of vertex displacement after ablation of cell bonds indicated in (I). Mean and s.e.m. are shown (n as in I). n.s. not significant.

A transcriptional response to Notch signaling is required for the separation of dorsal and ventral cells [35]. To further address the role of a transcriptional response to Notch signaling at the DV boundary, we tested whether activation of Notch signal transduction in a clone of cells would result in smooth clone borders. Smooth clone borders are an indication that cells inside the clone and outside the clone minimize contact, as is the case for cell populations interfacing at a compartment boundary [37]. Transduction of the Notch signal upon ligand binding involves the cleavage of the Notch receptor and release of the intracellular region of Notch, which binds to Suppressor of Hairless (Su(H)) to induce the expression of target genes [38,39,40]. Expression of the intracellular region of Notch (N^intra^) is sufficient to induce Notch target gene expression in the absence of ligand [38]. We used Flp-mediated recombination [41] in conjunction with the Gal4-UAS system [42] to express N^intra^ from a transgene in marked clones of wing disc cells. Flies lacking the *N*^*intra*^ transgene served as controls. Expression of N^intra^ resulted in the expression of Wingless, a Notch target gene, inside the clone cells (S1C and S1D Fig). Control clones and clones expressing N^intra^ were generated and analyzed three days after Flp-mediated recombination. Control clones showed a coherent appearance and irregular outlines (Fig 3A,3C and 3E). Clones expressing N^intra^ also had a coherent appearance, but were less irregular and often displayed a more roundish shape compared to control clones (Fig 3B,3D and 3F). We quantified the shape of the clones by a geometric measure termed ‘clonal roughness’. Clonal roughness describes the variance of the distance of a boundary from a curved line as a function of the length *L* of the analyzed boundary segment [14] (Fig 3G). The dependence of clonal roughness on segment length L describes the scaling behavior of the clone interface. Clones of cells expressing N^intra^ had a significantly reduced clonal roughness compared to control clones for all segment lengths analyzed, except for L = 1 average cell bond length where clonal roughness was undistinguishable (Fig 3H). These data show that local activation of Notch in clones of cells is sufficient to lead at medium and large scales to smooth clone borders.

### Expression of N^intra^ in clones of cells does not increase cell bond tension

To test whether clonal activation of a transcriptional response to Notch signaling can influence cell bond tension, we generated control clones and clones expressing N^intra^. Three days after Flp-mediated recombination, we ablated cell bonds along the clone borders and inside clones expressing N^intra^. The resulting vertex displacements were quantified. Final vertex displacements were similar upon laser ablation of cell bonds at the border of control clones and upon laser ablation of cell bonds inside clones expressing N^intra^ (Fig 3I). For laser ablation of cell bonds at borders of clones expressing N^intra^ vertex distance also initially increased, but the vertices rapidly retracted reaching a distance similar to the one prior to laser ablation after around 50 seconds (Fig 3I). The initial velocities of vertex displacements were indistinguishable for cell bonds at borders of control clones and clones expressing N^intra^, and for cell bonds inside clones expressing N^intra^ (Fig 3J). Thus, activation of a transcriptional response to Notch signaling is under the tested conditions not sufficient to increase cell bond tension.

### Expression of Capricious in clones of cells reduces clonal roughness

**Fig 4:**
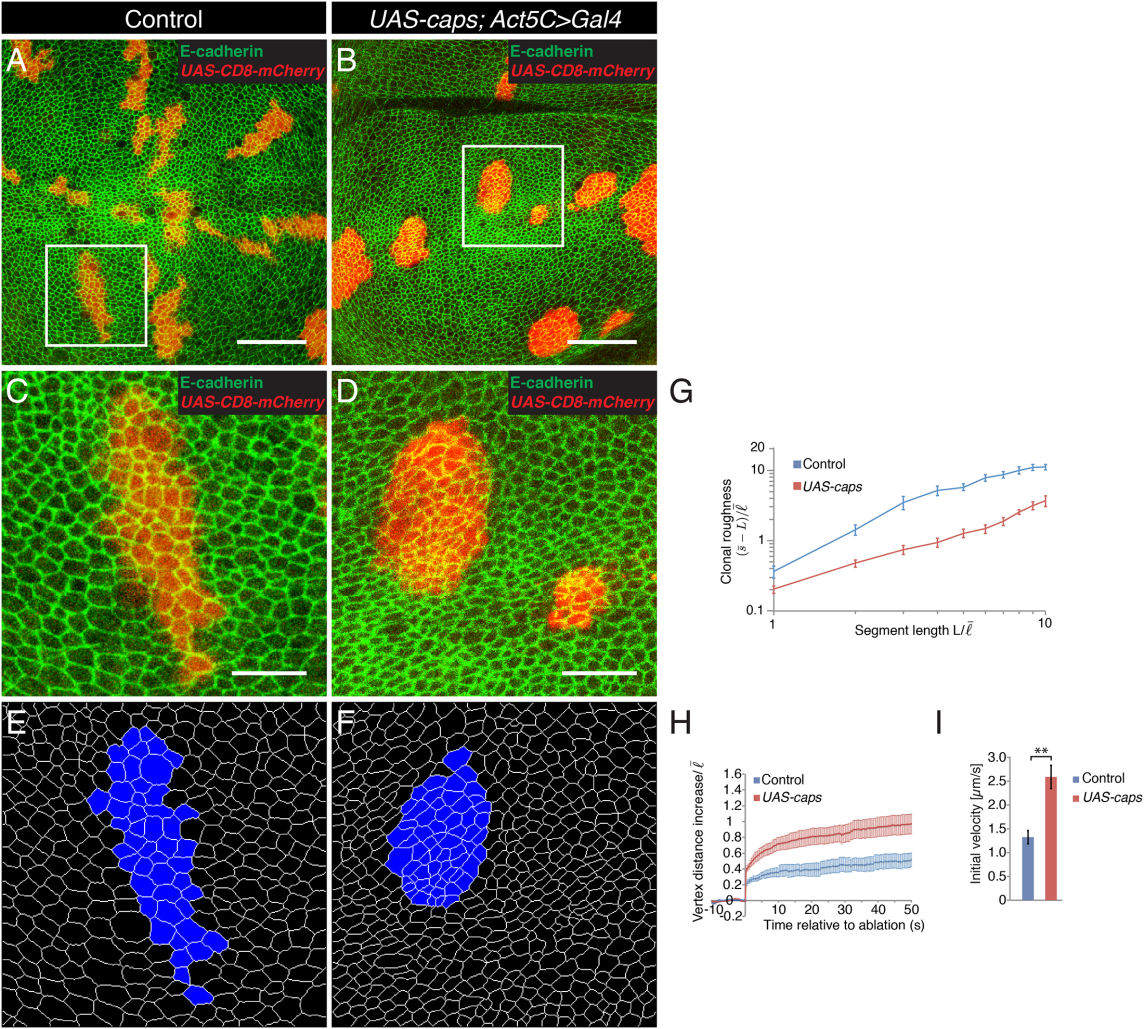
Expression of Capricious in clones of cells leads to smooth clone borders and increased cell bond tension at the clone border. (A,B) Wing discs of wandering stage third instar larvae displaying control clones (A) or clones expressing Capricious (B, *Act5C>Gal4*, *UAS-caps*) marked by the expression of CD8-mCherry (red) and stained for E-cadherin (green). Scale bars: 30 μm. (C,D) Magnification of the inset in (A,B). Scale bars: 10 μm. (E,F) Segmentation of the images shown in (C) and (D). The clones are marked in blue. (G) Clonal roughness for control clones and clones expressing Capricious. Segment length and clonal roughness values are normalized by the average cell bond length in the tissue $\bar{l}$ = 1.7 μm. Mean and s.e.m. are shown (control, n = 6 wing discs; mutant, n = 4 wing discs), *P* <0.001–0.05 for L = 3–10. (H) Change in distance *d* between the vertices at borders of control clones or of clones expressing Capricious after ablation (normalized to $\bar{l}$) as a function of time. Mean and s.e.m. are shown (control, n = 7; *caps*, n = 11 cuts). (I) Initial velocity of vertex displacement after ablation of cell bonds indicated in (H). Mean and s.e.m. are shown (n as in H). ** *P*<0.01.

Expression of Capricious and Tartan during mid third instar larval development is transiently confined to the cells of the dorsal compartment under control of Apterous [24]. Clones of cells doubly mutant for *capricious* and *tartan* do not disturb the shape of the DV boundary [24]. However, overexpression of Capricious or Tartan in the dorsal compartment of *apterous* mutant wing discs partially restores a straight DV boundary shape [24]. It has been proposed that the differential expression of Capricious and Tartan between dorsal and ventral cells contributes to the straight shape of the DV boundary [24]. To further analyze whether a difference in Capricious expression can contribute to the straight DV boundary shape, we expressed Capricious in clones of cells and analyzed the morphology of the clone borders three days after Flp-mediated recombination. Clones expressing Capricious had smooth borders and an overall roundish shape [24,43](Fig 4A–4F). Clonal roughness of clones expressing Capricious was significantly reduced compared to control clones for all segment lengths *L* analyzed, except for L = 1–2 average cell bond length where clonal roughness was undistinguishable (Fig 4G). Thus, the differential expression of capricious is sufficient to lead to smooth-edged and roundish clone borders consistent with the view that the differential expression of Capricious in wild-type wings discs contributes to the characteristic straight DV boundary shape.

### Expression of Capricious in clones of cells increases cell bond tension along clone borders

The expression of Capricious and Tartan exclusively in the dorsal compartment during mid third instar larval development correlates with a local increase in cell bond tension along the DV boundary [24,28]. To test whether the differential expression of Capricious between dorsal and ventral cells can contribute to the local increase in cell bond tension along the DV boundary, we ablated cell bonds along borders of control clones and of clones expressing Capricious and analyzed the resulting vertex displacements. Final vertex displacements and initial velocities of displacement were increased when cell bonds at borders of clones expressing Capricious were ablated compared to when borders of control clones were ablated (Fig 4H and 4I). The initial velocity of vertex displacement was increased approximately twofold (Fig 4I). These data show that differences in Capricious expression between two cell populations can contribute to a local increase in cell bond tension at the interface between the cell populations.

### Theoretical analysis of the influence of cell bond tension on shaping clones of cells

Our analysis above showed that clones expressing Capricious had smooth borders, a lower clonal roughness compared to control clones and an approximately two-fold increased cell bond tension along the clone border. To explore the influence of cell bond tension on clone shape, we performed numerical simulations of tissue growth using a vertex model [33]. The vertex model describes the adherens junctional network of epithelia as a network of polygons. It takes into account physical parameters that characterize the mechanical properties of cells and cell bond tension. In our simulations, we used initial configurations containing 16 cells. We introduced cell clones by assigning one cell of the initial configuration as the founding cell of a clone. The growth of the tissue was simulated by introducing stochastic cell divisions at constant rate. Each cell division resulted in the doubling of cell area and the formation of a new bond through the center of the cell. The junctional network was remodeled to satisfy local force balances. Our simulations thereby create configurations of clones with changing shapes. The size of clones after five generations was determined and the shape of clones comprising 30, 60, and 100 cells was characterized by clonal roughness.

**Fig 5:**
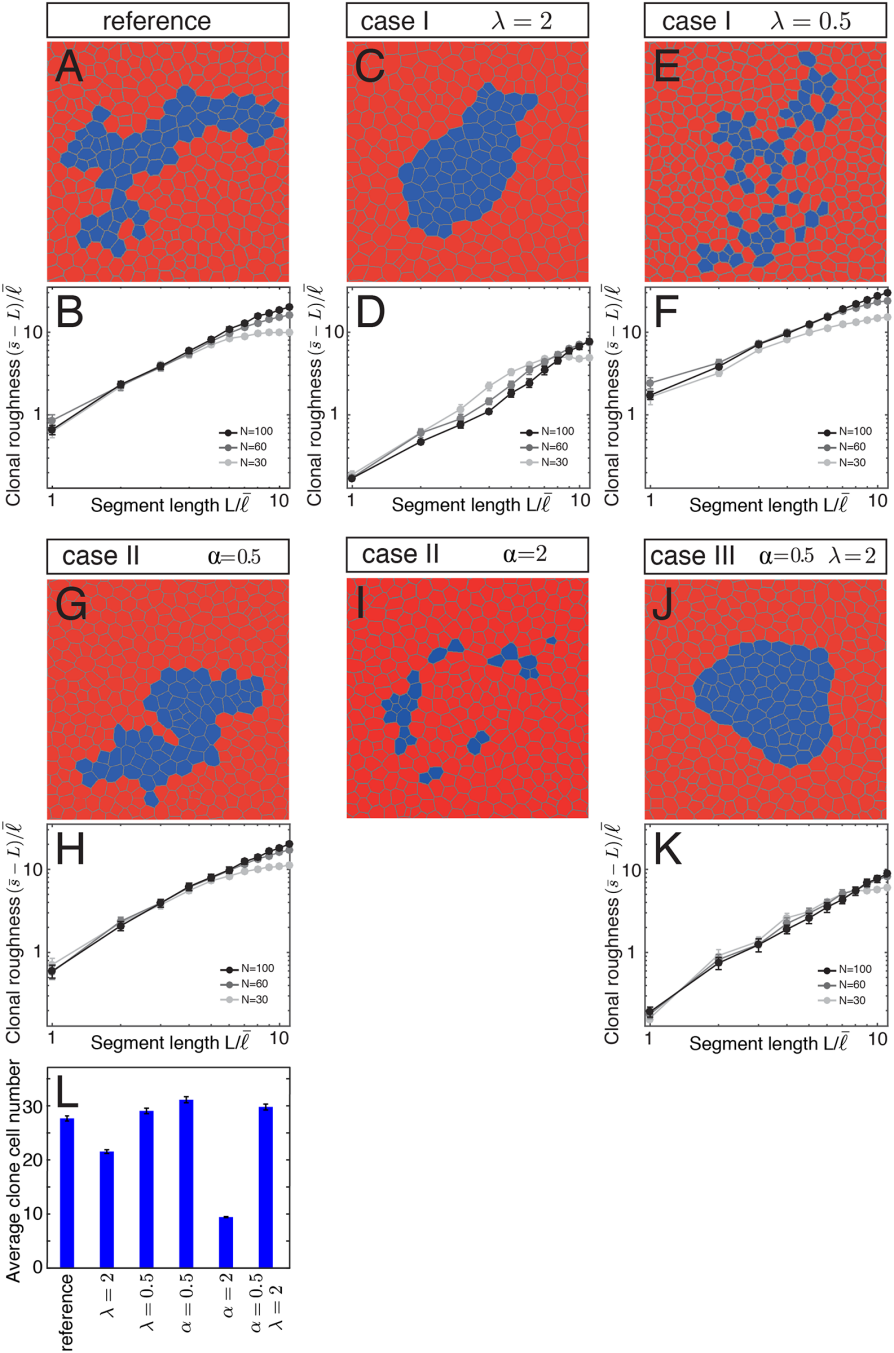
Influence of cell bond tension on clone shape. (A-K) The first and third rows represent examples of final configurations of networks of cell bonds of a clone (blue) in a tissue. The second and fourths rows depict clonal roughness for clone sizes N = 30, 60, and 100 cells as a function of segment length *L*. Segment length and clonal roughness values are normalized by the average cell bond length in the tissue $\bar{l}$. Mean and s.e.m. are shown (n = 30 realizations for each case and parameter value). (A,B) Reference case: All cells and cell bonds have the same properties. (C-F) Case I: Cell bond tension at the clone border is increased by a factor λ = 2 or decreased by a factor of 2 (λ = 0.5). (G-I) Case II: Cell bond tension in all cells of the clone is increased by a factor α = 2 or decreased by a factor of 2 (α = 0.5). (J,K) Case III. Cell bond tension at the clone border is increased by a factor λ = 2 and cell bond tension in all cells of the clone is decreased by a factor of 2 (α = 0.5). (L) Average clone cell number after five generations for the reference and the cases shown in (C-K). Mean and s.e.m. are shown (n = 2500 realizations for each case and parameter value).

We first considered a reference case, where cell bond tension was the same on all cell bonds. In this case, each clone formed a coherent group of cells similar to experimental control clones in the wing disc (Fig 5A). Clone shape was irregular resembling the shape of experimental clones. Clonal roughness increased with segment length *L* (Fig 5B), as was the case for experimental control clones. In case I, we altered cell bond tension along the clone border compared to cell bond tension elsewhere by a factor λ. For λ = 2 (i.e. cell bond tension is twofold higher at the clone border compared to elsewhere), clone size was slightly reduced compared to the reference case and clones had a roundish shape and smooth appearance (Fig 5C and 5L). Clonal roughness was reduced compared to the reference (Fig 5D). For λ = 0.5 (i.e. cell bond tension is twofold lower at the clone border compared to elsewhere), clone size was comparable with the reference, but clones had a more irregular shape and a dispersed appearance compared to the reference (Fig 5E and 5L). Single clone cells were surrounded by non-clonal cells. Clonal roughness was increased compared to the reference (Fig 5F). In case II, we altered cell bond tension for all cells inside the clone compared to elsewhere by a factor α. When cell bond tension inside the clone was half of the cell bond tension elsewhere (α = 0.5), clone size was similar to the reference and clones had a coherent appearance and their shapes were irregular (Fig 5G and 5L). Clonal roughness was comparable to the clonal roughness of reference clones (Fig 5H). When cell bond tension inside the clone was twice the cell bond tension elsewhere (α = 2), the clones did not grow to high cell numbers and the few cells that were detectable did not form a coherent clone but rather patches of one to few cells (Fig 5I and 5L). Clonal roughness could not be measured in this scenario. In case III, we combined a twofold higher tension at the clone border with a twofold lower tension inside the cells of the clone (λ = 2, α = 0.5). Clone size was similar to the reference and clones had a roundish shape and a smooth appearance (Fig 5J and 5L). The clonal roughness was reduced compared to the reference and similar to clones where only cell bond tension was twofold increased at the clone border (λ = 2) (Fig 5K). We conclude that local differences in cell bond tension can have various influences on the size and shape of the clone. A local decrease in cell bond tension along the clone border results in clone fragmentation. An increase of cell bond tension in all cells of the clone results in fewer clonal cells and clone fragmentation. A local increase in cell bond tension along the clone border results in smooth-shaped clones.

## Discussion

The spatiotemporal regulation of mechanical tension is important for epithelial morphogenesis. Here, we have addressed the mechanisms that locally increase cell bond tension along the DV boundary of *Drosophila* wing imaginal disc epithelia. We show that the selector gene *apterous* and the signaling molecule Notch are both critically required for increased cell bond tension along the DV boundary and for the characteristic straight shape of this compartment boundary. Moreover, we show that transcriptional activation of the Notch signaling pathway on its own is not sufficient to increase cell bond tension. Mosaic expression of the early Apterous-target gene *capricious*, however, suffices to increase cell bond tension at clone borders. Finally, we use theory to show that patterned mechanical forces can have various influences on clonal size and shape in epithelia.

### The role of Apterous and Notch for increasing cell bond tension and DV boundary shape

The selector gene *apterous* plays an important role in establishing and maintaining the DV boundary [17]. Apterous plays at least two independent functions with regard to boundary formation. First, during early third instar development, the time around which the DV boundary is established, Apterous induces the expression of the two leucine-rich repeat proteins Capricious and Tartan in dorsal cells [24]. Capricious and Tartan have been proposed to provide dorsal cells with a ‘dorsal’ affinity. Second, by inducing the expression of Fringe, Apterous indirectly leads to the activation of Notch signaling in a strip of cells centered on the DV boundary. Notch, in part, contributes to the formation of the DV boundary by repressing the miRNA *bantam* [44]. Notch further contributes to the formation of the DV boundary by regulating F-actin assembly and/or Myosin II motor activity. Both F-actin and Myosin II levels are transiently elevated at cell junctions along the DV boundary during early third instar larval development. These elevated amounts of F-actin and Myosin II require Notch function [31,36]. F-actin and Myosin II have been proposed to form an actomyosin cable that acts as a ‘fence’ preventing the mixing of dorsal and ventral cells [31,36].

Our previous work showed that cell bond tension along the DV boundary is increased as compared to the bulk of the tissue from mid—to—late third instar larval development [28]. We show here that the increase in cell bond tension along the DV boundary both requires Notch and Apterous function. Notch signaling and Apterous function are not per se required for cell bond tension, since cell bond tension in the bulk of the tissue is not affected in *N*^*ts*^ or *apterous* mutants. We propose that the apposition of Apterous expressing (dorsal) and non-Apterous expressing (ventral) cells elicits the increase in cell bond tension along the DV boundary.

How does the apposition of Apterous expressing and non-Apterous expressing cells induce an increase in cell bond tension along the DV boundary? The apposition of Apterous expressing and non-Apterous expressing cells leads to the induction of Notch signaling. However, it is difficult to explain how Notch signaling could increase cell bond tension specifically at the DV boundary, because the Notch signal is transduced and elicits target gene expression on either side of the DV boundary. Moreover, Notch activation can be spatially uncoupled from the DV boundary [45], indicating that activation of Notch is not sufficient to define the DV boundary (but see [36]). Consistent with this view, we find that expression of N^intra^ is not sufficient to increase cell bond tension. Taken together, these data argue in favor of a permissive, rather than instructive, role of Notch signaling in increasing cell bond tension along the DV boundary.

The role of Notch signaling at the DV boundary thus differs from the role of Hedgehog signaling at the AP boundary with respect to regulating cell bond tension. A difference in Hedgehog signal transduction is both necessary and sufficient to increase cell bond tension at the AP boundary [14]. This discrepancy likely arises from the fact that Notch signals bi-directionally across the DV boundary, whereas Hedgehog signaling is unidirectional from posterior to anterior cells and therefore generates a difference in anterior and posterior cells that is exploited to locally increase cell bond tension at the AP boundary. Bidirectional signaling across the DV boundary may have evolved because dorsal and ventral compartments give rise to apposed wing surfaces, and therefore must be of identical size and shape. This might be most easily achieved by bidirectional signaling across a symmetrically positioned compartment boundary [6]. These circumstances, however, necessitate additional mechanisms to confine the increased cell bond tension to the DV boundary, which are elicited by the selector gene *apterous*.

A possible scenario how Apterous confines the increase in cell bond tension to the DV boundary is based on the two Apterous target genes *capricious* and *tartan*. During mid-larval development, around the time the DV boundary is established, Apterous induces the expression of Capricious and Tartan within dorsal cells [24]. We find that clonal overexpression of Capricious results in increased cell bond tension at the clone borders. This result suggests that a difference in Capricious expression between neighboring cells induces higher levels of cell bond tension at the interface between the two cell populations. This finding might explain the local increase of cell bond tension soon after the DV boundary has been formed, at a time when Capricious expressing cells abut non-Capricious expressing cells. However, this cannot explain the local increase in cell bond tension at later larval stages, since Capricious (and Tartan) expression is no longer confined to the dorsal compartment during these stages. Instead, both proteins are at this later developmental stage expressed in the lateral part of the wing disc pouch, where they participate in short-range signals mediating cell survival [43].

### Influence of cell bond tension on clone size and shape

Force balances on junctional bonds determine cellular network topology in epithelia [33]. We have used simulations of tissue growth to determine the influence of patterns of cell bond tension on clone shape and size. Our results show that a local increase in cell bond tension at clone borders suffices to decrease clonal roughness (Fig 5C and 5D), consistent with previous reports [30]. Interestingly, neither a local decrease in cell bond tension at the clone border (Fig 5E and 5F), nor a bulk increase in mechanical tension on all bonds within the clone decreases clonal roughness (Fig 5I). These findings further support the hypothesis that a local increase in cell bond tension is important to lead to smooth boundaries between cell populations [30].

Interestingly, a decrease in cell bond tension at the clone borders had the opposite effect and increased clonal roughness. The interface shape between cell populations is determined by junction remodeling and cell-cell intercalations [29]. Previous work showed that a local increase in tension biases cell-cell intercalations to maintain smooth borders. Cell-cell intercalations are biased by the asymmetric shrinkage of junctions that is determined by unequal tension on the cell bonds participating in the cell intercalation. A local decrease, similar to a local increase, in tension will lead to unequal tension on cell bonds participating in cell intercalation and thus bias cell-cell intercalations to promote cell mixing. This suggests that cell mixing accounts for the observation that, in the simulations, reduced cell bond tension at the clone border promotes clone fragmentation.

Clone fragmentation is a hallmark of cell competition [46], a process where slow growing ‘loser’ cells are eliminated by faster growing ‘winner cells’ (reviewed in [47,48]). A recent study showed that cell competition requires cell mixing and that differential-growth and higher tension at winner-winner cell bonds as compared to loser-loser cell bonds promote cell mixing [49]. Our simulations indicate that in addition to these bulk differences in cell bond tension between two cell populations also local differences in cell bond tension at the interface between two cell populations can promote cell mixing. We did not observe that cell number was reduced in clones that had a decreased cell bond tension at the clone border, suggesting that cell mixing *per se* may not suffice for cell elimination.

Increasing cell bond tension in the bulk of the clone led in our simulations also to clone fragmentation. In this case, however, the number of clone cells was strongly reduced, suggesting that clone cells were eliminated. Cell elimination is promoted by differences in interface contractility between two cell populations [50]. Our observation indicates that also differences in bulk mechanical properties of cells can drive cell elimination. An increase in bulk mechanical tension on cell bonds in the clone may alter force balances inside the clone, which have been suggested to influence cell elimination [33]. Increased bulk tension inside the clone may also recapitulate features of cell crowding, which has been shown to promote cell elimination [51].

### A mechano-biochemical process shapes the DV boundary

We propose a model in which the integration of selector gene and signal pathway activities with patterns of mechanical tension contributes to the shaping of the DV boundary. The difference in the expression of the *apterous* selector gene between dorsal and ventral cells elicits a local increase in cell bond tension along the DV boundary that biases cell intercalations to prevent mixing of dorsal and ventral cells. In part, the local increase in cell bond tension is elicited through Apterous’s function to induce Notch signaling in a strip of cells on either side of the DV boundary. Notch signal transduction is required to increase cell bond tension along the DV boundary, however, a second Apterous dependent function is likely required to confine the increased mechanical tension to cell bonds along the DV boundary. This second function needs to be further explored, but may involve in early third instar stage Capricious and Tartan.

This work, together with our previous findings [14], suggests that one common mechanism to induce cell bond tension along a compartment boundary is to appose cells with different selector gene or signal transduction activities. It will be interesting to learn in the future how cells perceive this difference and respond to it by modulating their mechanical tension.

## Materials and Methods

### Fly stocks

The following fly stocks were used: *ap*^*UGO35*^ [23], *ap*^*Gal4*^ [52], *UAS-GFP*, *N*^*ts*^, *Act5C>CD2>Gal4* [53], *UAS-N*^*intra*^ [54], *ubi-DE-cad-GFP* [55], *UAS-CD8-mCherry* [29], and *UAS-caps* [56]. The fly stocks were reared on cornmeal-soy-malt-yeast food at 25°C unless otherwise noted. For experiments involving *N*^*ts*^ flies, animals were raised at 18°C before shifting to 29°C for 48 hours prior to dissection in late third instar larval stage.

### Clonal analysis

To induce expression of FLP recombinase (under control of a heat-shock promoter), larvae were subjected to a 17 min. heat-shock at 37°C 72 hours prior to dissection in late third instar larval stage.

### Antibody staining

Wing discs were dissected, stained and fixed according to standard protocols. Primary antibodies used were rat anti-DE-cad (DCAD2, Developmental Studies Hybridoma Bank (DSHB) 1:50) and mouse anti-Wingless (4D4, DSHB, 1:100). Secondary antibodies were donkey anti-rat CY5 IgG (H+L) (Jackson ImmunoResearch Laboratories, 1:200) and anti-mouse Alexa 633 (Life Technologies, 1:200).

### Image acquisition, processing and analysis

Image stacks of fixed wing discs were acquired on Zeiss LSM780 or Leica SP5 confocal microscopes with 40X/1.4 NA and 63X/1.2 NA objectives. Image stacks were projected and the resulting image was segmented based on the E-cadherin channel [57]. The roughness of the compartment boundary and clonal roughness was analyzed as described previously [14]. Clones larger than 40 cells were used for the analysis.

### Laser ablation

Laser ablation experiments were performed as previously described [30]. An inverted microscope with a 63x/1.2 NA water immersion objective equipped with a pulsed, third harmonic solid-state UV-laser (355 nm, 400 ps, 20mJ/pulse) was used. Adherens junctions were identified using E-cadherin-GFP (*ubi-DE-cad-GFP*). The DV boundary and clones of cells were identified by GFP (*ap-Gal4*, *UAS-GFP*) and CD8-mCherry (*Act5C>Gal4*, *UAS-CD8-mCherry*), respectively.

### Simulation of clones in growing tissues

We describe the mechanics of a tissue using a vertex model [33]. In this model, balanced network configurations are given as local minima of a work function

$$\text{E}=\frac{\text{K}}{2}\overset{{\text{N}}_{\text{c}}}{\underset{\alpha =1}{\sum }}{\left({\text{A}}_{\alpha }-{\text{A}}^{\left(0\right)}\right)}^{2}+\underset{\langle \text{ij}\rangle }{\sum }\Lambda_{\text{ij}}\;l_{\text{ij}}+\frac{\Gamma }{2}\overset{{\text{N}}_{\text{c}}}{\underset{\alpha =1}{\sum }}{\text{L}}_{\alpha }{}^{2},$$

where A_α_ is the area of cell α, A^(0)^ is the preferred area and *K* denotes cell area elasticity. *ℓ*_ij_ is the length of a cell bond, < ij > between two adjacent vertices i and j, and Λ_ij_ describes the mechanical tension of the corresponding bond. The perimeter of cell α is L_α_ and Γ denotes the perimeter elasticity. In this picture, the mechanics of networks is characterized by two dimensionless parameters $\bar{\Lambda }=\Lambda /(\text{K}{\left({\text{A}}^{\left(0\right)}\right)}^{\frac{3}{2}},\bar{\Gamma }\;=\;\Gamma /\left({\text{K A}}^{\left(0\right)}\right)$.

We consider the situations where cell bond tension is given by ${\bar{\Lambda }}_{ij}={\bar{\Lambda }}_{0}$ for all cell bonds outside the clone, ${\bar{\Lambda }}_{ij}={\bar{\Lambda }}_{C}$ for all cell bonds inside the clone, and ${\bar{\Lambda }}_{ij}={\bar{\Lambda }}_{B}$ for the cell bonds along the clone border. We introduce the relative cell bond tension of the clone by $\alpha ={\bar{\Lambda }}_{C}/{\bar{\Lambda }}_{0}$ and the relative cell bond tension along the clone border by $\lambda =2{\bar{\Lambda }}_{B}/\left({\bar{\Lambda }}_{0}+{\bar{\Lambda }}_{C}\right)$.

Tissue growth is simulated by introducing stochastic cell divisions. During each simulation step a cell division occurs, for that one cell is picked up randomly and divides in a random direction. We then minimize the work function to find the new balanced configuration of the cellular network.

The clone initiates from one cell in a hexagonal network of 16 cells at generation *G* = 0. Cells within the clone divide into two daughter cells belonging to the same clone. The shape of a clone changes due to remodeling of cellular junctions when cell divisions occur. We consider the reference case where cell bond tension is constant for all cells, α = 1 and λ = 1. In the other cases cell bond tension inside the clone or at the clone border is increased or decreased given by indicated factors α and λ when clone size is bigger than 8 cells. In our simulations we average clonal roughness for the clones with the same size.

### Statistical analysis

Statistical analysis was done using a two-sample, unpaired Student’s *t*-test.

## Supporting Information

S1 Fig Wingless expression in *N*^*ts*^ wing discs and clones expressing N^intra^. (A,B) Wing discs from control and *N*^*ts*^ mutant larvae reared for 48 h at 29°C and expressing GFP (red) in the dorsal compartment (*apGal4*, *UAS-GFP*) were stained for Wingless (cyan). Scale bars: 30 μm. (C,D) Wing discs displaying control clones (C) or clones expressing N^intra^ (D, *Act5C>Gal4*, *UAS-N*^*intra*^) marked by the expression of CD8-mCherry (red) and stained for Wingless (cyan). Scale bar: 30 μm.
(TIF)
