# Supplementary figures and images for: The Selector Gene apterous and Notch Are Required to Locally Increase Mechanical Cell Bond Tension at the Drosophila Dorsoventral Compartment Boundary
Source: PLoS One. 2016 Aug 23;11(8):e0161668. doi: 10.1371/journal.pone.0161668 (PMC4995041; doi:10.1371/journal.pone.0161668)

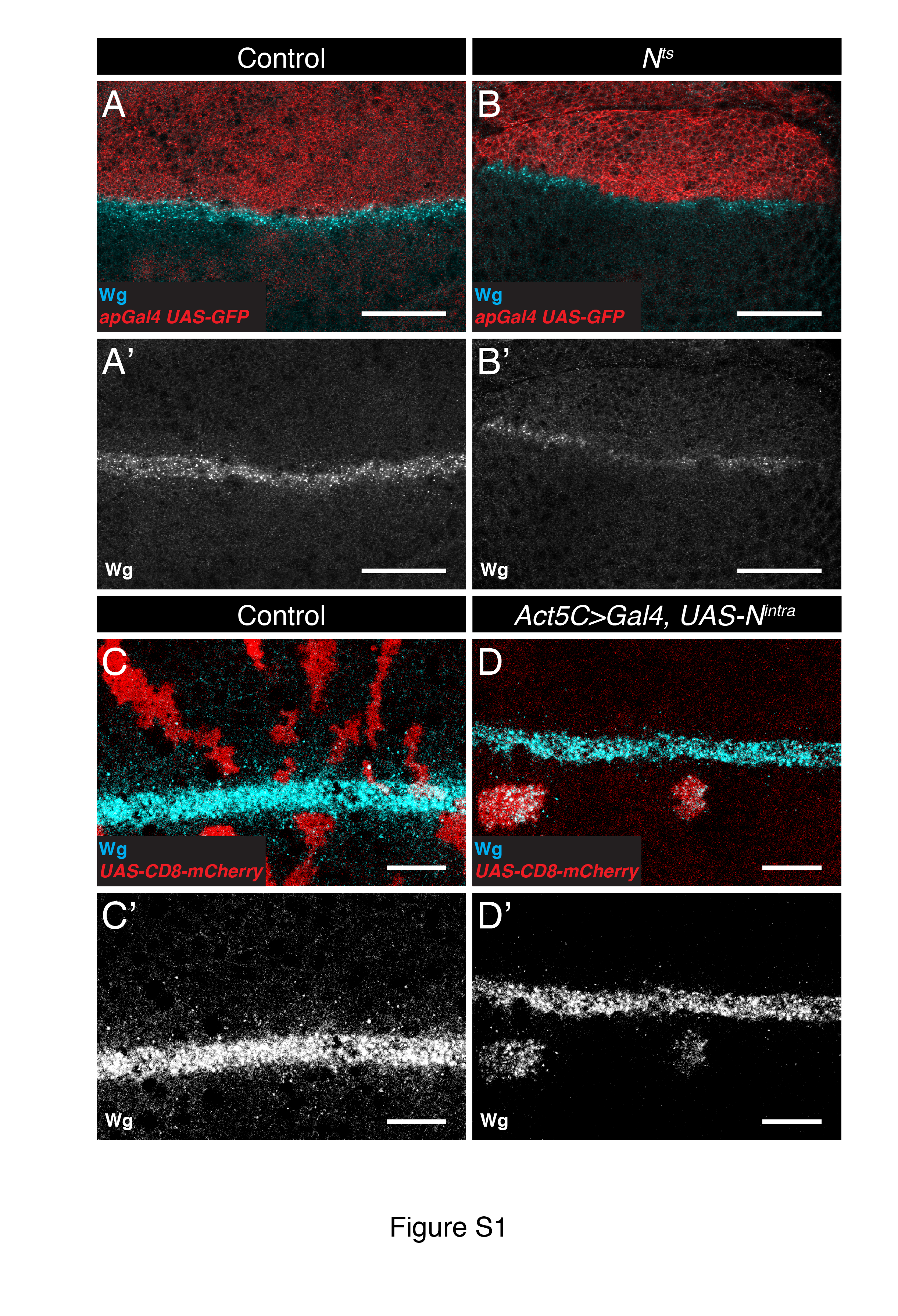

Supplement: S1 Fig — (A,B) Wing discs from control and Nts mutant larvae reared for 48 h at 29°C and expressing GFP (red) in the dorsal compartment (apGal4, UAS-GFP) were stained for Wingless (cyan). Scale bars: 30 μm. (C,D) Wing discs displaying control clones (C) or clones expressing Nintra (D, Act5C>Gal4, UAS-Nintra) marked by the expression of CD8-mCherry (red) and stained for Wingless (cyan). Scale bar: 30 μm. (TIF) [file pone.0161668.s001.tif]
